# The Value of Three-Dimensional Modeling on Repositioning and Soft Tissue Filling in Microsurgical Reconstruction of Secondary Mandibular Defects: A Retrospective Study

**DOI:** 10.7759/cureus.22336

**Published:** 2022-02-17

**Authors:** Ioannis Tilaveridis, Glykeria Pantazi, Gregory Venetis, Konstantinos Ditsios, Christos Angelopoulos, Konstantinos Antoniades

**Affiliations:** 1 Oral and Maxillofacial Surgery, Aristotle University of Thessaloniki, Thessaloniki, GRC; 2 Plastic and Reconstructive Surgery, General Hospital “George Papanikolaou”, Thessaloniki, GRC; 3 Orthopaedics, General Hospital “George Papanikolaou”, Thessaloniki, GRC; 4 School of Dentistry, Kapodestrian University of Athens, Athens, GRC; 5 Oral and Maxillofacial Surgery, General Hospital “George Papanikolaou”, Thessaloniki, GRC

**Keywords:** fibula, reconstruction, secondary, mandibular defect, 3d printing

## Abstract

Purpose

Secondary mandibular defect reconstruction is a challenging operation. It is performed rather rarely, as in most of the cases a primary reconstruction is preferable. Restoration of function and symmetry, in secondary reconstruction of mandibular defects, requires accurate repositioning of segments and appropriate soft tissue filling. The purpose of this study is to estimate the value of three-dimensional (3D) digital modeling to meet the above requirements, in secondary microsurgical reconstruction of mandibular defects.

Methods

Five cases of mandibular defects, with various degrees of asymmetry and dysfunction, were restored secondarily, with fibula flaps, after virtual reconstruction on a 3D model derived from their computed tomography, with the utilization of CAD-CAM software. Software reproduced symmetrical 3D models by mirroring the healthy side. Occlusion was restored by fine adjustments on 3D models and finally a reconstruction plate was pre-bent on them, prior to its sterilization for use in surgery. Three out of the five cases received an osteo-cutaneous flap, while in the other two patients, an osseous flap was used. Lower face symmetry, mouth opening, and main patient’s complaints were evaluated pre- and postoperatively to assess the value of the 3D modeling.

Results

All flaps survived. Hematoma occurred in two patients and was resolved after evacuation. In two patients, a fistula observed, was attributed to screw loosening, and treated after a surgical debridement and screw replacement. Intraoral exposure in the posterior part of the fibula flap was recorded in one patient, possibly from wound dehiscence due to tension of the intraoral tissue, and successfully covered with an ipsilateral nasolabial flap. The resulting symmetry and function were satisfactory in all the patients.

Conclusion

Secondary mandibular defects are often related with cosmetic disfigurement, misalignment of mandibular segments, and mandibular malfunction. For the correction of mandibular continuity, symmetry, and restoration of function, preoperative 3D modeling may be an important tool, according to our results.

## Introduction

In resection of tumors involving the mandible, immediate reconstruction of mandibular defects is the first choice [1]. Secondary reconstruction, at a later stage, is an alternative option for a minority of cases, especially in advanced oral cancer and in complications related to infection, stress-fracture of osteo-synthetic materials, plate exposure, or lack of expertise [2]. Secondary mandibular defect reconstruction is a challenge for the surgeon, as many difficulties can arise and should be addressed. Difficulties are often related to postsurgical misalignment of mandibular segments that are distorted and rotated away from their natural position due to muscle or scar contraction, making the secondary reconstruction more challenging [3]. Another important issue, after postoperative radiotherapy, is the poor quality of soft tissues and the alteration of recipient vessels at the irradiated neck that necessitates the inspection of their patency [4]. 

Cosmetic disfigurement due to the lack of fullness and asymmetry of the lower third of the face is a major complaint in most of the cases, when primary reconstruction of the mandible fails or is performed without bone grafting, leaving a deep concavity at the site of bone defect [3,4]. Isolated plates bridging the gap between stumps of a segmental mandibulectomy, without bone grafting, run the risk of plate stress fracture, losing accurate orientation of mandibular segments. In some cases, failure of conventional regional or local flaps can result in intraoral or extraoral plate exposure. Removal of the plate becomes then necessary, deteriorating asymmetry and function [5-7]. 

Correction of distortion of the mandibular stumps, for restoration of symmetry and reestablishment of dental occlusion, may be assisted by printed three-dimensional (3D) models of the mandible. A 3D model of the mandible helps diagnostically, because by holding the jaw model in their hand, surgeons can estimate with accuracy the form and dimensions of the defect. Digital reconstruction of the affected site by mirroring the healthy side produces a virtual corrected model of the mandible. On this second, corrected model, a plate is prepared to fit the contour of the virtually corrected side. This can significantly reduce the operating time and blood loss [3]. Virtual surgical planning offers to the surgeon the perspective of better understanding the complexities of the maxillofacial skeleton and thus the ability to plan more demanding operations [8].

Free vascularized bone grafts have a lot of advantages over simple bone grafts, especially in oncologic patients [9]. Fibula free flap is the working horse for primary and secondary mandibular defect reconstruction. The longevity of the bone, the incorporation of skin in an osteo-cutaneous free flap, and the rigidity of the bone, capable to withstand osseointegrated dental implants, are some of the advantages of the fibula bone flap [10]. 

In this paper, we present five cases with secondary mandibular defects after resection of tumors of the oral cavity. In all cases preoperative 3D models of the mandible were used as part of the surgical plan. The aim of this study is to evaluate how the printed 3D mandibular models could facilitate the surgeon to perceive and measure the dimensions of mandibular defects, to accurately reconstruct distorted and rotated mandibular segments and to shorten the operation time by bending and conform the reconstruction plate over a printed model.

## Materials and methods

Six consecutive patients, with mandibular defects due to previous segmental mandibulectomies for tumor involving the mandible, were planned for fibula free flap reconstruction at the University Department of Oral and Maxillofacial Surgery of the General Hospital G. Papanikolaou between 2015 and 2018. One female patient was excluded from being candidate for fibula free flap, as an interruption of the peroneal artery in its initial part was observed in both of her legs. Four out of the remaining five patients, who were included in this study, were operated for squamous cell carcinoma with infiltration of the mandibular bone, and one patient was operated for the removal of a recurrent ameloblastoma (recurred one year after initial excision) (Table 1). The cancer patients received postoperative radiotherapy, for two of them in combination with chemotherapy. 

**Table 1:** Demographic and clinical data of the patients. SCC: squamous cell carcinoma; MRND: modified radical neck dissection.

Patient no.	Birth year	Gen der	First operation/etiology	Location/stage TNM	Intermediate operation	Postoperative radiotherapy
1	1982	Male	2010/SCC	Mandibulectomy-right MRND/T4N1MX	2015/Plate removal	Yes
2	1955	Female	2016/SCC	Central and left lateral mandibulectomy - Bilateral MRND/T4N2cMx	2016/Plate removal	Yes
3	1961	Male	2015/SCC	Left mandibulectomy - MRND	2018/Plate exposure	Yes
4	1999	Male	2016/Ameloblastoma	Left mandibulectomy for recurrence of ameloblastoma	No need for intermediate operation	No
5	1964	Male	2012/SCC	Right mandibulectomy	2014/Change of reconstruction plate	Yes

**Figure 1:**
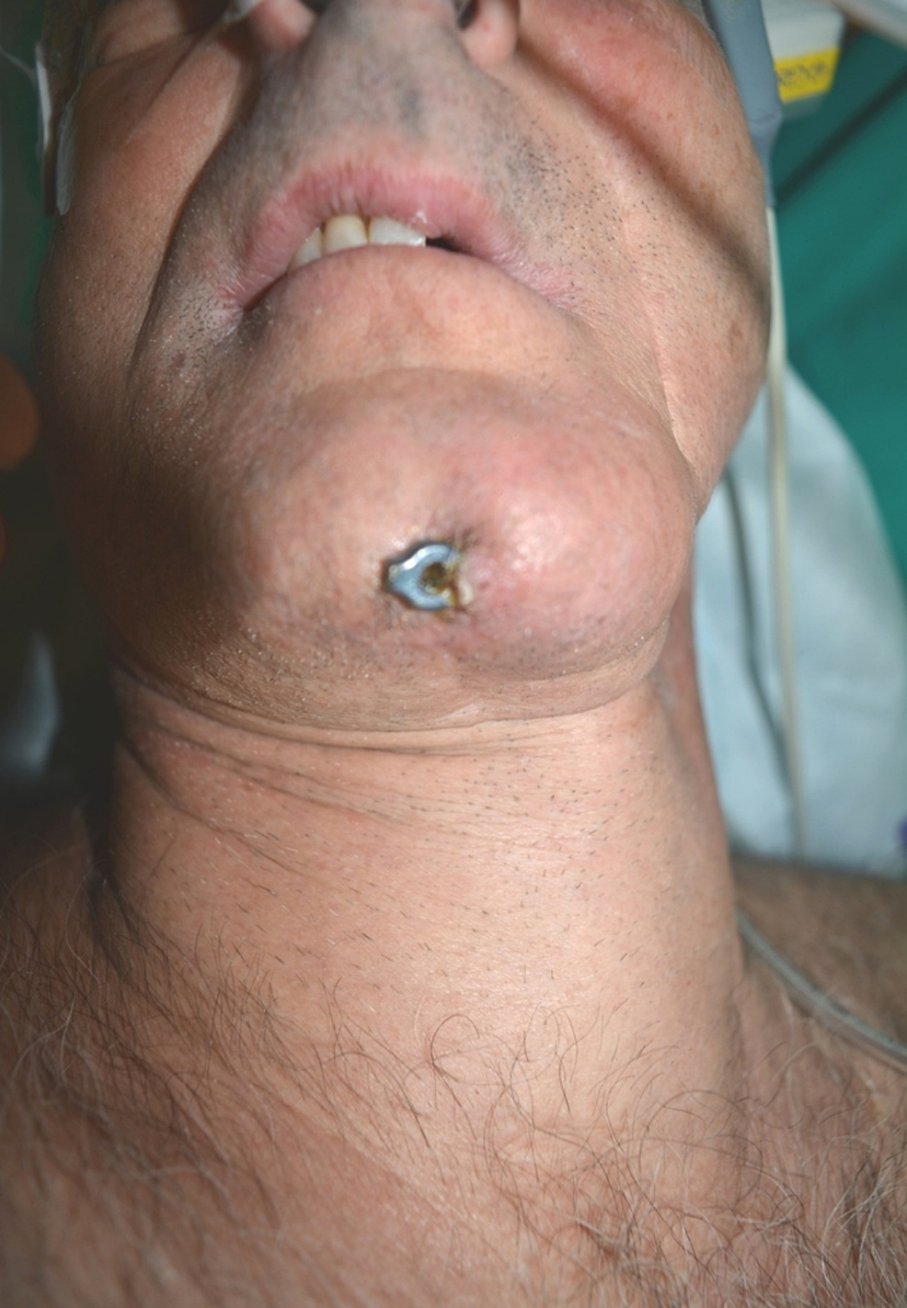
Exposure of the anterior part of reconstruction plate at the chin area.

The primary reconstruction of mandibular defects had been performed with locoregional flaps and a reconstruction plate to retain the mandibular shape. In one patient, the reconstruction plate was removed, postoperatively, because of infection and the mandibular segments remained without any bridging. Extraoral exposure of the reconstruction plate was observed in another patient (Figure 1). All the patients received fibula free flap.

**Figure 2:**
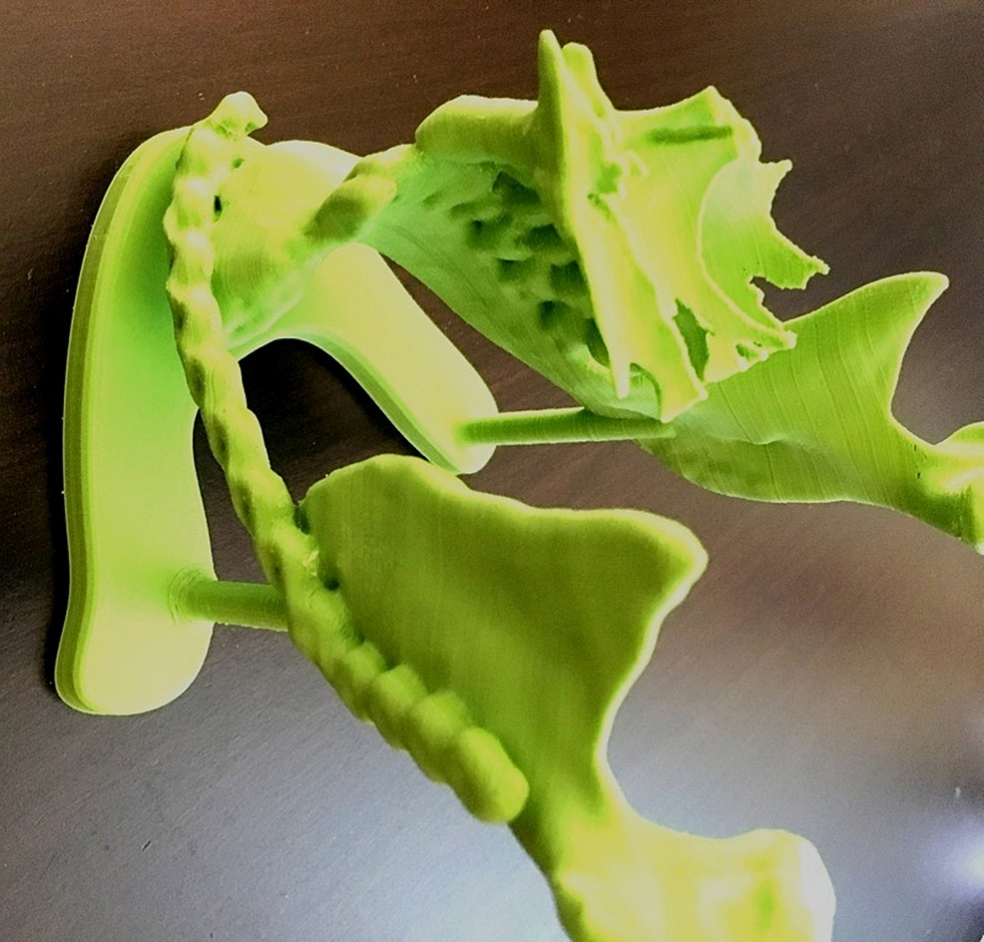
Three-dimensional model for estimation of the bone defect in the mandible.

The dimensions of the defects were calculated on the preoperative CT or cone beam computed tomography (CBCT) scan and re-estimated on its 3D printed model and a reconstruction plate was adapted preoperatively to the corrected mandibular segment to aid the reconstructive procedure (Figure 2). The 3D printed model was generated from the CTs, with the use of an ULTIMAKER 3 (EXTENDED), Zaltbommel, the Netherlands. The fused deposition modeling technique and poly-galactic acid material from the same manufacturer were used.

For the two patients with previous plate removal, displacement of the segments required the printing of a second 3D model for virtual correction of displacement with “mirroring” of the healthy hemi-mandible to replicate the missing segment. Asymmetry of the lower third of the face was measured clinically at two different extraoral sites and at the corresponding sites on CT or CBCT. These sites were defined by drawing a straight line along the midline of the face and neck and measuring the distances of points corresponding to the mandibular angles at the right and left sides.

**Figure 3:**
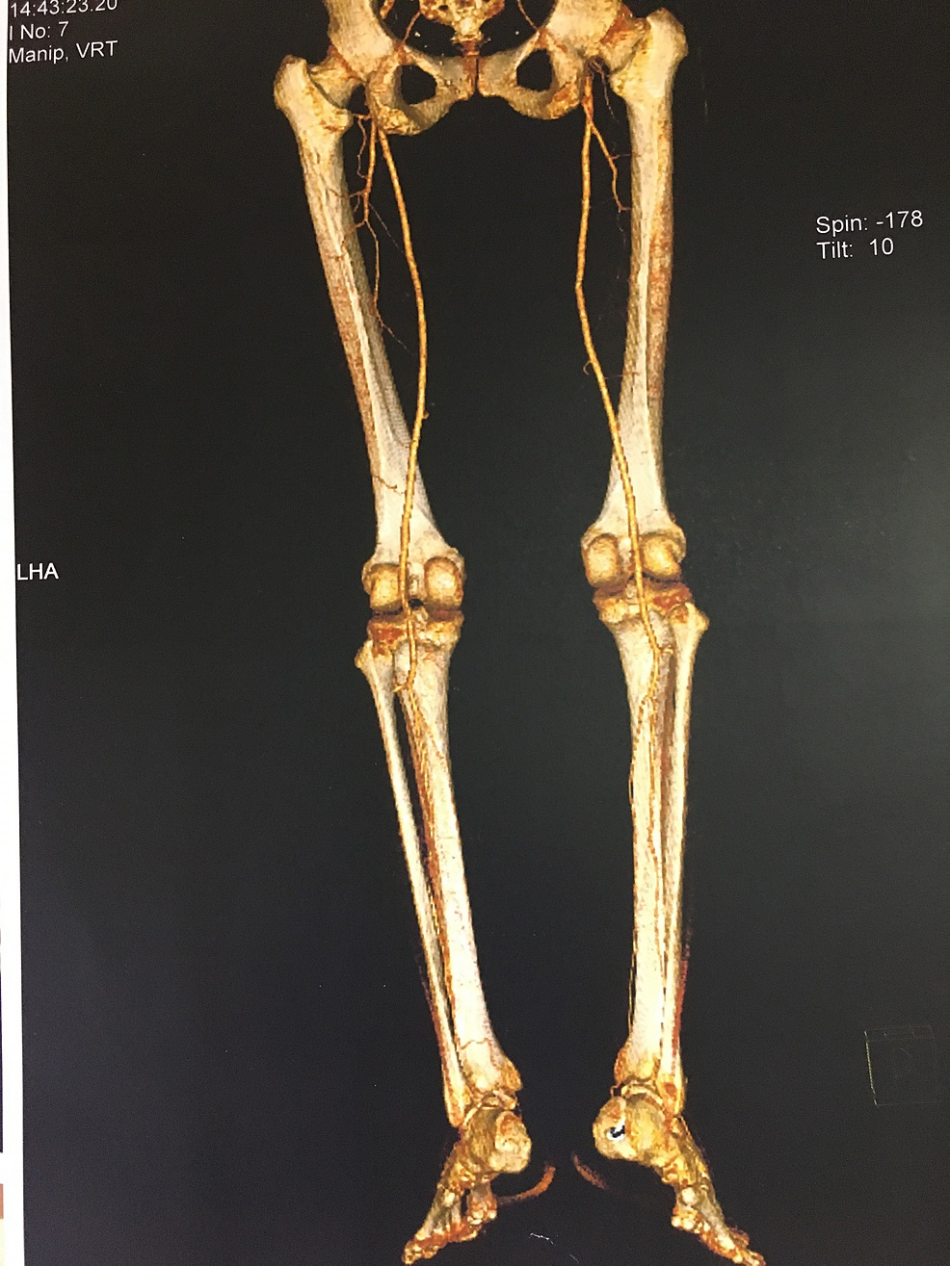
CT angiography showing the typical vascularity of the lower extremities. CT: computed tomography.

**Figure 4:**
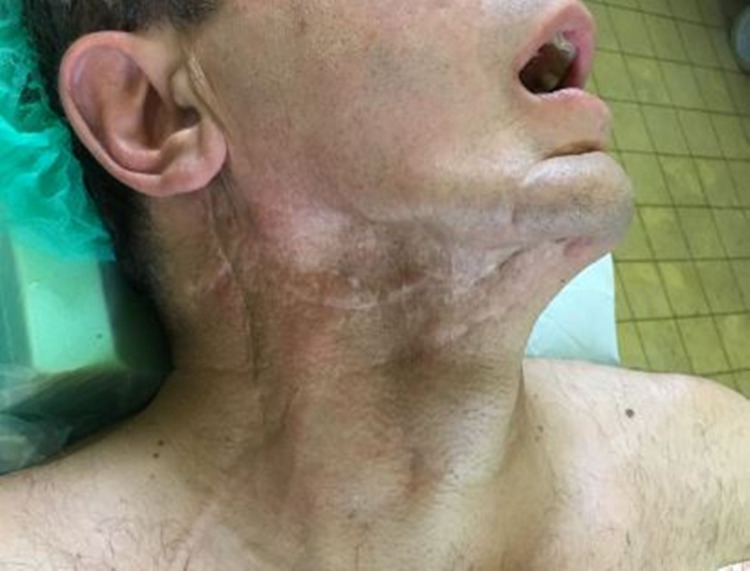
Hollowing of the submandibular area after neck dissection.

**Figure 5:**
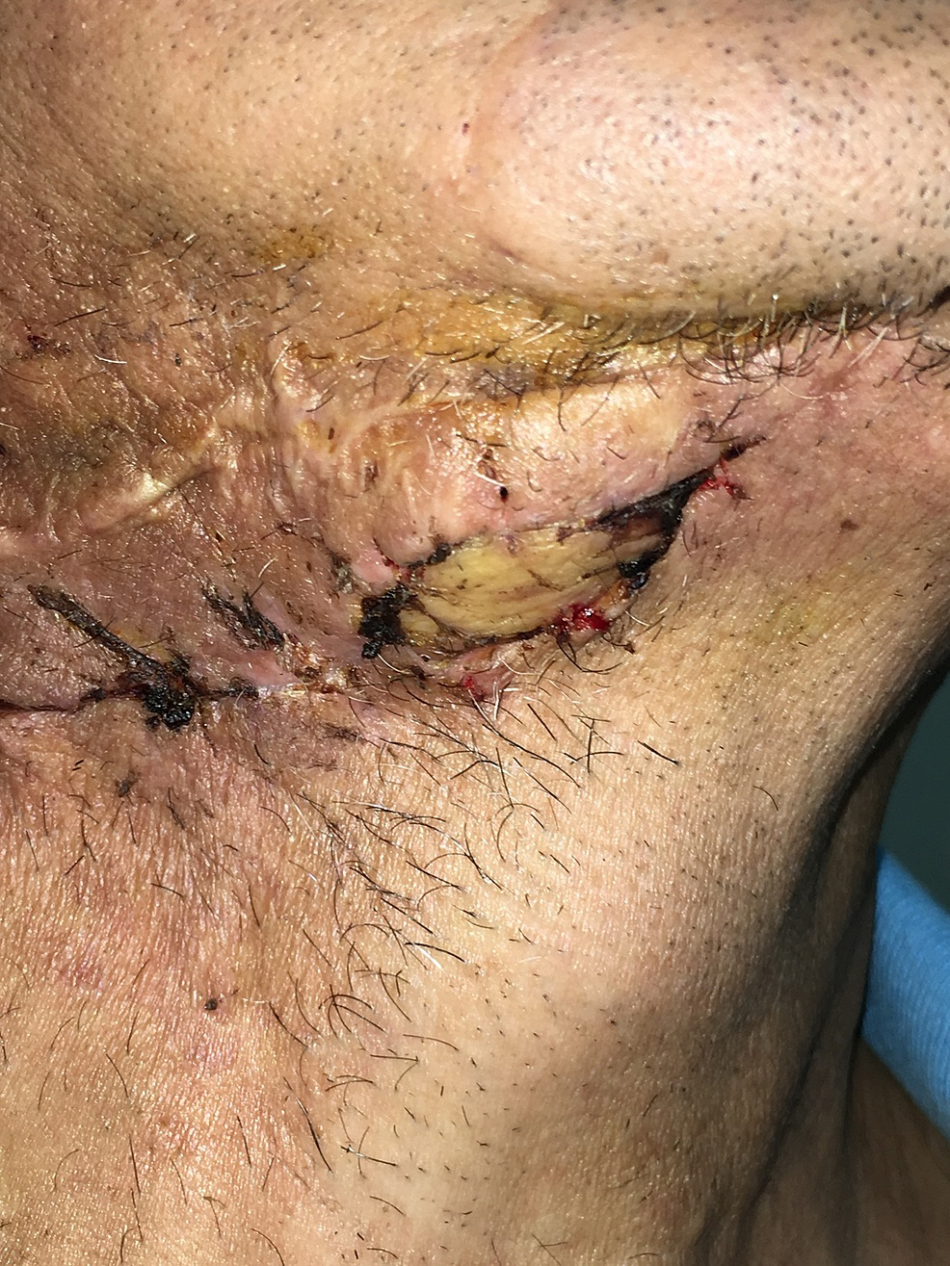
Fullness of the submandibular area, in the patient in Figure 4, with de-epithelialized skin of the fibula free osteo-cutaneous free flap. A small skin island of fibula flap remained in the submandibular area for flap monitoring.

A CT angiography was performed in all patients to investigate the vascular anatomy of the lower extremities and the recipient vessels of the neck (Figure 3). In one patient, a significant cosmetic issue was a gap (hollow) at the right submandibular area after neck dissection and subsequent radiotherapy. A draft estimation of the soft tissue defect was done in this case, by studying the 3D reconstruction of the face from the CT, to harvest an osteo-cutaneous fibula flap, de-epithelialize most of the skin - except from an island serving to monitor flap viability - and place it subcutaneously, for reconstruction of lost tissue volume at the submandibular area (Figures 4, 5).

**Figure 6:**
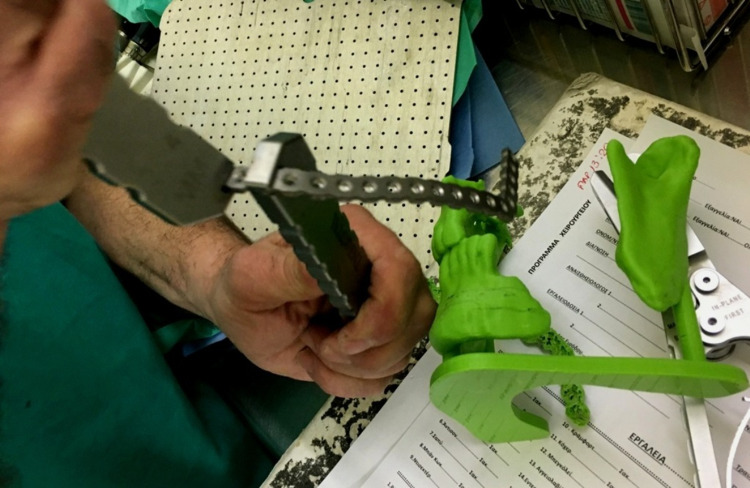
Preoperative shaping of the reconstruction plate for adaptation to the corrected three-dimensional model.

Virtually reconstructed 3D models were used as a guide for pre-bending of the reconstruction plates, to minimize the intraoperative time. The reconstruction plate was pre-shaped along the external surface of the printed/reconstructed model and was sterilized the day before the operation (Figure 6). During the operation, correction of the distorted segments of the mandible was performed and the pre-bent reconstruction plate was adjusted. After inspection of the accurate contact between the plate and the mandible, the defect’s dimensions were re-estimated and compared to the 3D printed models. Then, osteotomies of the fibula were performed, according to the preoperative schedule, with the aid of the 3D model and the guidance of the plate. In three of the patients, a suitable size of skin was collected with the bone and used for coverage of defects extraorally, intraorally, or subcutaneously. The follow-up was ranged from two to five years. Postoperatively, the survival of the flap, complications, and parameters such as the symmetry, the intergonial distance, bone healing at fibula edges, mouth opening, as well as the patient satisfaction were recorded.

## Results

The mean time of the flap harvesting was 2-3 hours and the anastomotic time ranged between 1 and 1.5 hours. Considering the preparation of the recipient site performed at the same time by a second team, the total operating time was in average 5 hours. Early postoperative complications (Table 2) included hematoma in two patients that was resolved after evacuation, without any negative effect on flap viability. 

**Table 2:** Management of postoperative complications.

Patient no.	Patient complaints	Reconstructive operation	Complications	Treatment of complications	Status of the flap
1	Esthetic disfigurement/functional disturbance	Left osteo-cutaneous fibula	Hematoma at the early postoperative period	Evacuation of hematoma	Viable
2	Esthetic disfigurement/functional disturbance	Right osteo-cutaneous fibula	Small intraoral exposure of the fibula	Coverage with nasolabial flap with local anesthesia	Viable
3	Extraoral projection of the plate's esthetic and functional disturbances	Right osseous fibula	Extraoral fistula	Curetage - removal of loose screw, plate trimming	Scintigraphy confirmed survival of the flap
4	Reduced mastication ability	Right osseous fibula	Intense hematoma at the early postoperative period	Evacuation	Viable
5	Esthetic and functional disturbances	Right osteo-cutaneous fibula	No complication	-	Viable

**Figure 7:**
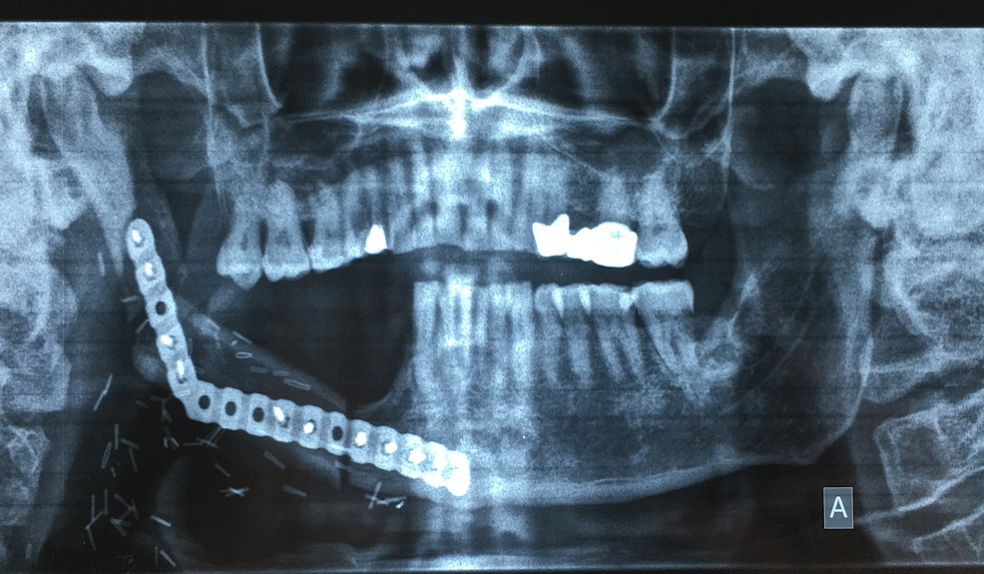
Orthopantomogram showing the adaptation of the fibula to the right mandibular defect.

**Figure 8:**
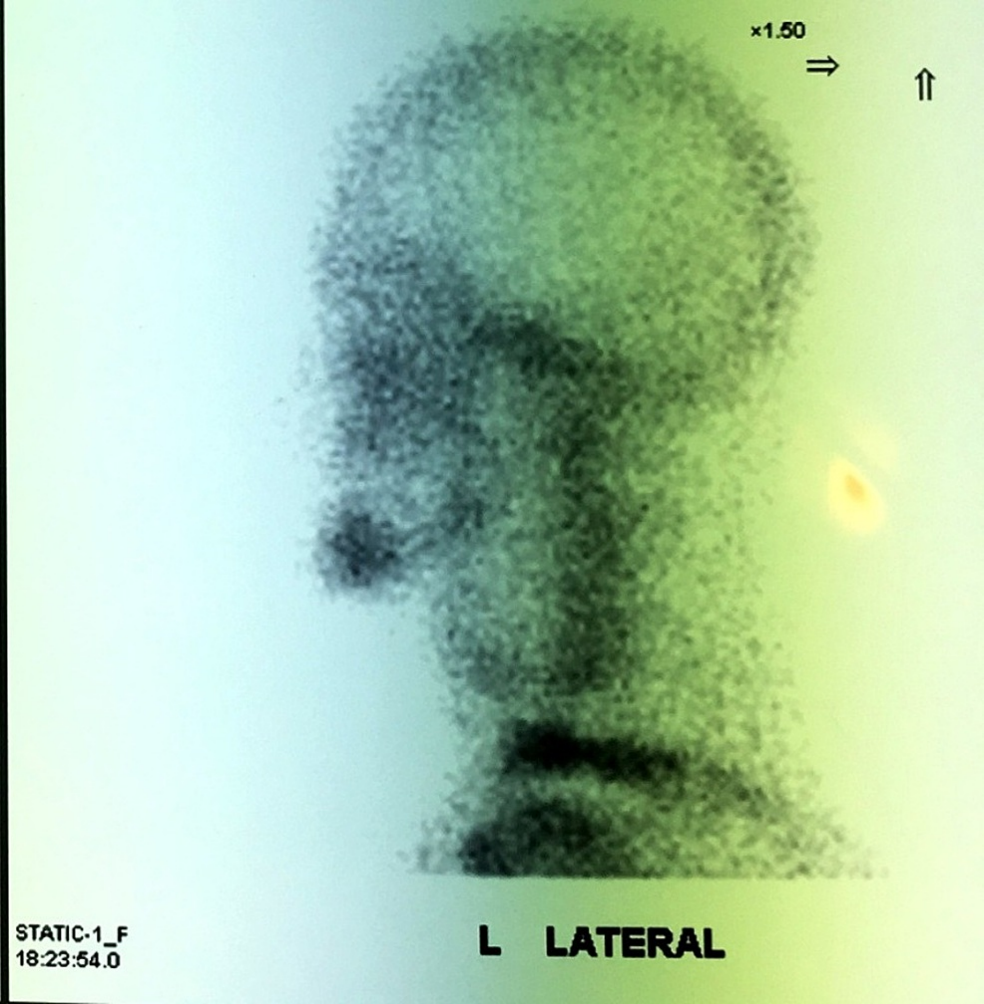
Technetium scintigraphy shows viability of the fibula.

Extraoral fistula was observed in two patients along with a local inflammation some months after the operation, necessitating examination of the wound. In both cases it was attributed to screw loosening without any influence on flap survival. In one of these patients the final screw and the distal edge of the plate were exposed through the fistula. Under the suspicion of flap necrosis, a scintigraphy was performed in this case, which confirmed the viability of the flap. Intraoral exposure in the posterior part of the fibula flap was recorded in another patient, one month postoperatively, possibly from wound dehiscence due to stress of the intraoral tissue. A nasolabial flap was used under local anesthesia to effectively cover the intraoral exposure of the fibula bone. All the flaps survived as confirmed by the survival of incorporated skin, orthopantomograms, and in one patient with technetium bone scintigraphy (Figures 7, 8).

**Figure 9:**
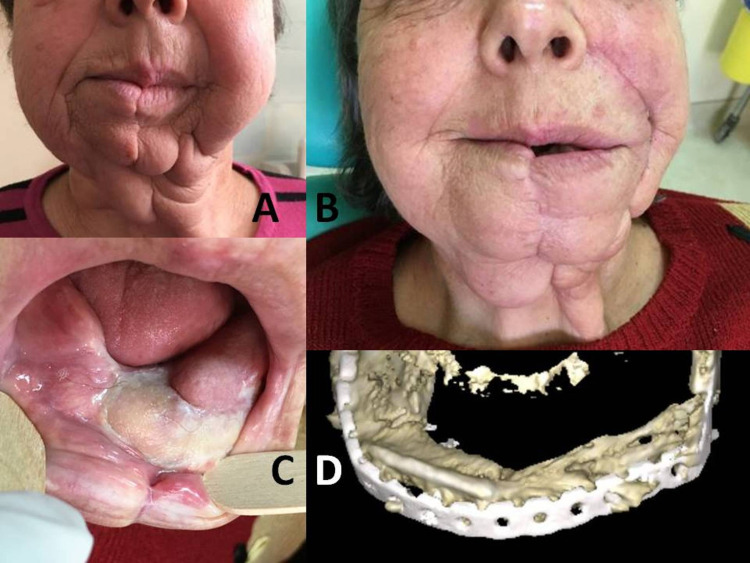
A: Face disfigurement after mandibulectomy for squamous oral carcinoma and early removal of the plate due to infection. B: Postoperative appearance of the patient after fibula flap reconstruction. C: Intraoral view of the skin part of the fibula osteo-cutaneous flap. Satisfactory mouth opening. D: Postoperative 3D CT, showing the reconstructed mandible. 3D: three-dimensional; CT: computerized tomography.

Restoration of occlusion of the existing teeth was performed in four patients. One patient was edentulous. Active mouth opening was also estimated postoperatively, and it ranged between 29 and 38 mm . Symmetry was satisfactory, compared to the preoperative situation (Figure 9). The remaining functions of the oral cavity, such as deglutition, swallowing, and speech, were also improved. All the patients were satisfied with the cosmetic and functional results (Table 3).

**Table 3:** Functional postoperative results. D: distal; P: proximal; +++: complete; ++: acceptable; +: poor bone healing at fibula edges; CT: computed tomography; CBCT: cone beam computed tomography.

Patient no.	Symmetry: distance of the mandibular angle from midline, in CT		Mouth opening (active)	Bone healing at fibula edges (estimation with CBCT)	Patient satisfaction
	Right	Left			
1	39 mm	44 mm	34 mm	D++, P+++	Complete
2	42 mm	38 mm	29 mm	D++, P+	Complete
3	47 mm	40 mm	32 mm	D+, P++	Complete
4	45 mm	42 mm	38 mm	D++, P+++	Complete
5	44 mm	41 mm	32 mm	D++, P+++	Complete

## Discussion

Mandibular defect reconstruction, performed usually as primary and rarely as secondary reconstruction of the mandible, is the mainstay of successful functional and cosmetic results [11,12]. According to some authors, conventional flaps, despite a lot of disadvantages compared to free vascularized flaps, are better to consider in cases where lengthy operations are not well withstood by patients with co-morbidities, or because of the increased cost of free flaps in comparison to the pedicled flaps [13]. In addition, in resource-constrained countries, or because of lack of expertise, primary reconstruction of the mandible with reconstruction plates bridging mandibular segments usually replaces free flaps [2,4]. In these cases, failure of a conventional regional or local flap can result in intraoral or extraoral plate exposure that necessitates plate removal. Even though soft tissue reconstruction may heal well, the lack of mandibular continuity leads to severe facial disfigurement. Fracture of the plate is another complication occurring months or years after exposure of the plate to masticator forces. Fracture of the reconstruction plate makes removal and replacement of the plate necessary [14,15]. The complications in the early or late postoperative period may compromise the reconstruction. Four out of our five cases had problems with the reconstruction plate.

Premature removal of reconstruction plates, but without immediate replacement due to flap necrosis and infection, creates additional difficulties because of intense tissue shrinkage and scar formation. Muscle contraction after plate removal leads to loss of orientation of the mandibular segments and collapse of dental occlusion, making the reconstruction of the mandible more complex and demanding for the surgical team [3]. Postoperative radiotherapy in most of oncologic patients with advanced tumors is another important factor for tissue contraction, making tissue shrinkage worse and affecting the situation of recipient vessels, if a free flap reconstruction is contemplated [3,16]. Four of our patients received postoperative radiotherapy, which along with modified neck dissection may compromise not only the quality but also the existence of available recipient vessels for the anastomosis. The convex shape of the mandible, the contraction of soft tissue, the distortion and displacement of mandibular ramus, the collapse of occlusion, and the irradiation of recipient vessels of the neck form an intricate mixture of problems to be solved in a successful secondary mandibular defect reconstruction [3].

New technology with virtual treatment planning, 3D printing, and customized plate construction provides a valuable therapeutic aid for contemplation and scheduling of the operation. 3D printing was a revolution in implant dentistry, as accuracy and precision are the mainstay of dental implantology [17]. Many articles in literature describe the application of 3D modeling in facial reconstruction [18,19]. However, there are few articles about microsurgical reconstruction of secondary mandibular defects [20] and even fewer using 3D digital models. Virtual reconstruction on a 3D model offers the opportunity of precise representation of the mandible and accurate correction of mandibular segments along with restoration of occlusion in dentate patients. In this model, the surgeon also can measure the exact size of the bony defect and estimate the lines of osteotomy for better adaptation of the flap. In the reality of the operating room, a difficulty sometimes exists with mobilization of mandibular stumps as they are stuck by muscle contraction and need wide mobilization. This difficulty, and the intra-operative adjustment of the reconstruction plate cumulatively lengthen the operating time, with the latest being responsible for an unpredictable symmetry of the lower face. Thus, probably the greatest benefit of virtual reconstruction on a 3D model is the prefabrication of the reconstruction plate, to achieve better symmetry and complete contact between the plate and the mandible [3,21,22]. In our series, 3D models contributed decisively to the reduction of the operating time as plate contouring in the operating room was avoided. The precontoured plate is also used as a navigator in cases with displacement of mandibular segments, helping to transpose them in the correct position.

Contraction of soft tissue, irrespective of its etiology, constrains surgeons when it comes to expansion of tissues and especially the intraoral tissue is sutured under tension. As a result, wound dehiscence may occur postoperatively. Extraoral release of contracted tissue is achieved in most of the patients and larger skin graft should be harvested with fibula flap to close the defect. Skin of the neck is harder and less pliable due to contraction and fibrosis after neck dissection and the additional radiotherapy [4]. To avoid this complication, a larger skin graft should be taken with the fibula flap. In one of our patients, poor quality of the covering soft tissue led to wound dehiscence and a nasolabial flap was needed to cover the intraorally exposed bone successfully. Soft tissue harvesting with the fibula flap is also useful to correct face asymmetry and disfigurement owing to the previous neck dissection and removal of a large quantity of the content of the submandibular triangle. This disfigurement in one of our patients was corrected by harvesting of appropriately scheduled skin graft with the fibula flap and subcutaneous placement after de-epithelialization, to increase the volume of the submandibular area.

Many studies highlight the need for preoperative angiography of the lower extremities before harvesting fibula free flap to identify possible congenital abnormalities or peripheral vascular disease that might pose danger to the vascularity of the lower extremities [23,24]. Angiography of the lower extremities in one female patient of our case series revealed lack of patency of the peroneal artery in both of her legs and she was excluded as candidate for fibula free flap harvest. The results of this study and others highlight the importance of preoperative imaging prior to fibular flap transfer to identify both acquired and congenital abnormalities in order to decrease the risk of vascular compromise to the lower extremity.

In secondary mandibular defect reconstruction, preoperative CT angiography of the neck is also considered necessary for surgeons to be better prepared for the difficulties they will encounter. Goetze et al. [25] refer the loss of two fibula free flap in seven patients and other researchers stress that most complications for this kind of flap involve vascular compromise [3,26]. Preoperative angiography or 3D CT images of the neck are mandatory to select the most suitable recipient vessels according to its location and diameter [3,27]. Inspection of location and repletion of recipient vessels before performing the anastomosis is mandatory, as many of these vessels, and especially arteries, are occluded at their peripheral end and the operated neck vessels are located more deeply. According to some authors, finding of these vessels is the first step in secondary mandibular reconstruction [4].

## Conclusions

In the new era of virtual simulation, virtual surgical planning, CAD/CAM, rapid-prototyping procedures, intraoperative navigation, and other complex technological improvements, training, certain dexterities, and probably expense are required. Despite the limitations of our study (among which the small number of cases and its retrospective mode), we concluded that 3D printed models offer the possibility to keep the mandible in the surgeon’s hand, to virtually correct the asymmetry with objective methods, to measure the extent of the defect with accuracy, to pre-bend the reconstruction plate and then to sterilize it before surgery or to make customized reconstruction plates, and to schedule the operation with safety.

Evolution of 3D modeling in reconstructive surgery led to development of more sophisticated and accurate techniques that include cutting guides both for the fibula and the mandible, achieving accurate adaptation of the fibula to a custom-made reconstruction plate. Future research may focus on predictability of results obtained from computer-aided novelties and on dissemination of e-planning skills. The latest can decrease the cost and multiply the benefit for patients, worldwide.
